# Commercial Hoverboard Reverse Engineering and Repurposing for a Stabilized Platform: A Recyclable Solution for Modular Robotic Bases

**DOI:** 10.3390/s25123833

**Published:** 2025-06-19

**Authors:** Antoine Leblanc, Lùka Tricot, Duncan Briquet, Mohamed Aziz Slama, Christophe Delebarre

**Affiliations:** School of National Institute of Applied Sciences—INSA Hauts-de-France, Campus Mont Houy, 59313 Valenciennes, France; antoine4246@gmail.com (A.L.); luka.tricot02@gmail.com (L.T.); duncanbriquet842@gmail.com (D.B.); azizslama26@gmail.com (M.A.S.)

**Keywords:** inverted pendulum, two-wheeled robot, hoverboard, control system, reverse engineering, robotics, mobile base, modular robotics

## Abstract

**Highlights:**

**What are the main findings?**

- Reverse engineering discarded hoverboards yields a low-cost, self-balancing robotic base.
- Paves the way for versatile, low-cost robot bases in healthcare and manufacturing.

**What is the implication of the main finding?**

- Supports eco-friendly, modular robotic systems through hardware reuse.
- Offers easily reconfigurable platforms for robotics.

**Abstract:**

Sustainability and resource optimization have spurred interest in giving a second life to used equipment, often discarded after limited use. Within this framework, we conducted a multidisciplinary, final-year engineering project to explore the reverse engineering and repurposing of commercial hoverboards for an auto-stabilizing, modular robotic platform, with emphasis on medical applications such as transporting medication. The innovation lies in recycling hoverboards to develop a teleoperated, stabilized base that can accommodate additional modules—for instance, a multifunctional arm or a transport shelf—akin to existing commercial robots. Our methodology involves disassembling and reprogramming the hoverboard’s motor controllers and sensors to maintain horizontal stability. Control is realized through the sensor fusion of accelerometer and gyroscope data, processed by a Kalman filter and implemented in a Proportional-Integral-Derivative (PID) loop. A user-friendly Human-Machine Interface (HMI), hosted on an ESP32 microcontroller, enables remote operation and monitoring. Experimental results show that the platform autonomously balances, carries payloads, and achieves high energy efficiency, highlighting its potential as a sustainable and versatile solution in modular robotic applications.

## 1. Introduction

Robotics is increasingly present in industry, healthcare, and domestic environments, driven by the need for efficient automated systems that can perform repetitive tasks and operate alongside humans [1,2]. However, the development of cost-effective and sustainable robotic platforms remains a key challenge, particularly when it comes to balancing performance requirements with environmental considerations. In this context, discarded electric personal transport devices—particularly hoverboards—represent a promising source of robust hardware that can be repurposed instead of relegated to waste streams.

### 1.1. Motivation: Low-Cost and Sustainable Solutions

Commercial hoverboards, widely available and often discarded after limited use, represent an untapped source of robust hardware for robotics. They are equipped with brushless DC motors, a lithium-ion battery with a battery management system (BMS), and integrated tilt or Hall sensors to achieve self-balancing functionality. One key factor behind their disposal is that the battery eventually loses sufficient capacity to transport an adult rider—often around 80 kg—across typical commuting distances [3]. Yet even a partially degraded battery can still power lighter robotic loads, providing an opportunity to recycle and repurpose these components into cost-effective, auto-stabilizing platforms. By salvaging not only the motors but also the existing driver boards, the approach can substantially reduce both material costs and electronic waste, aligning with environmental goals.

A self-balancing two-wheeled platform derived from hoverboards can serve as a versatile mobile base for transporting loads up to human weight and beyond, but now with a focus on modular design. In medical environments, for instance, such a platform could carry medication or tools through hospital corridors, assisting healthcare personnel. Similar applications in industrial settings might include autonomous tool trolleys or inspection robots, where maneuverability and minimal footprint are essential. Leveraging existing motors, sensors, and embedded boards exploits the robust engineering of consumer hoverboards, originally designed to handle daily vibrations and unpredictable user inputs.

### 1.2. Hoverboard Architecture and Components

**Figure 1:**
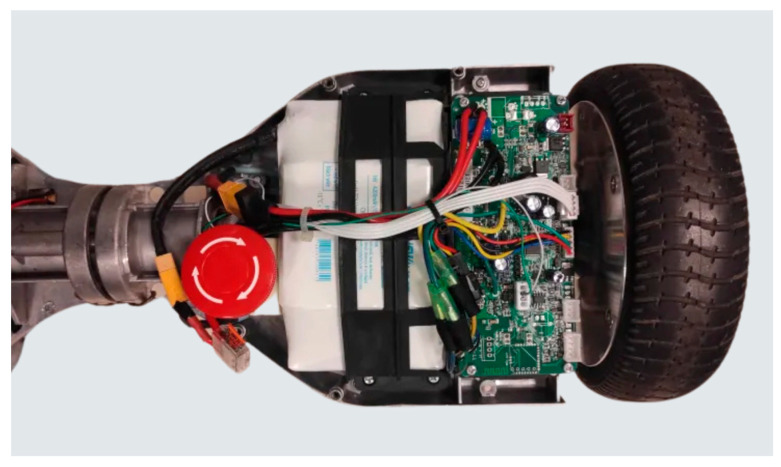
Photo of a hoverboard’s internal electronics.

In standard consumer usage, a hoverboard operates as an inverted pendulum on two wheels. Mechanically, it features a steel frame with a central pivot that ensures structural rigidity and helps distribute weight evenly across the wheels. Each wheel houses a brushless DC motor, typically rated around 250–350 W, along with tilt or Hall sensors that measure angular displacement and speed (Figure 1). These sensors feed data to the onboard mainboard (motherboard), which processes it in real time to adjust motor commands and maintain balance. The hoverboard is powered by a 36 V lithium-ion battery, monitored by an integrated BMS that oversees charging, discharging, and cell balancing. Additional elements often include IR footpads that detect user weight or foot position, LED lights for visibility, and a physical power switch. While these features are optimized for personal transportation, they can readily be repurposed for robotic applications once the battery degrades beyond standard ride requirements. The motor driver boards, in particular, can be reverse-engineered to allow external microcontroller control, bypassing the original tilt-based logic.

### 1.3. Challenges and Control Approaches for Two-Wheeled Platforms

The primary challenge of two-wheeled platforms lies in maintaining stability, as they inherently resemble an inverted pendulum system. Several stabilization methods have been proposed in the literature [4,5], including fuzzy logic control [6] and Proportional–Integral–Derivative (PID) control [7]. MPC offers advanced predictive capabilities but requires high computational resources, which may not be feasible for cost-effective systems. Fuzzy logic controllers excel in handling nonlinearities and uncertainties but demand extensive tuning and expertise [8]. Among these methods, PID control stands out as a robust and efficient solution due to its simplicity, adaptability, and ease of implementation. By continuously adjusting motor speeds based on error feedback from sensors, PID control ensures the real-time correction of imbalances, making it a practical choice for lightweight microcontrollers such as the ESP32 used in this project.

A two-wheeled platform has been chosen because of its agility and appropriateness for medical settings, where space and speed restrictions are crucial. PID control was selected to meet the project’s goals of developing a dependable and reasonably priced system with easily accessible resources. With this method, the hoverboard-based platform can be stabilized effectively while maintaining a computational overhead that is within the limits of the hardware currently in use. This novel use of two-wheeled mobile robots is explored in the following sections, along with its technological implementation, experimental validation, and future directions.

The present work explores how the essential subsystems of a commercial hoverboard—especially its motors and integrated driver boards—can be reverse-engineered and repurposed for a self-balancing robotic platform. Even when the battery cannot reliably power an 80 kg rider, its residual capacity often remains sufficient for lighter-duty robotics. The reuse of existing driver boards, sensors, and mechanical components helps lower costs and reduce the environmental footprint, thereby contributing to sustainable development initiatives. Building upon established research in two-wheeled balancing robots [9], the proposed approach demonstrates that hoverboards constitute a viable foundation for stabilized teleoperated mobile bases, provided that their control firmware can be adapted or replaced. This article details the reverse engineering methodology [10,11,12], the hardware and software modifications required, and the experimental results validating the feasibility of repurposed hoverboard platforms in various application scenarios.

## 2. Reverse Engineering Methodology

To implement the self-balancing system, we decided to repurpose key components from a commercial hoverboard. This allowed us to reduce costs while gaining access to powerful motors and control boards designed for balance management. Reverse engineering played an important role in repurposing the hoverboard into a two-wheeled robotic platform. The process began with disassembling the hoverboard to identify and understand the components essential for its operation, including the motor driver boards, the main control board, and the IMU sensors. Each element was tested individually to ensure functionality and compatibility with new control systems. One of the biggest challenges during this phase was the lack of detailed documentation on the hoverboard. With so little information available, it became necessary to rely on existing knowledge, testing different approaches, and learning through trial and error to find effective solutions.

**Figure 2:**
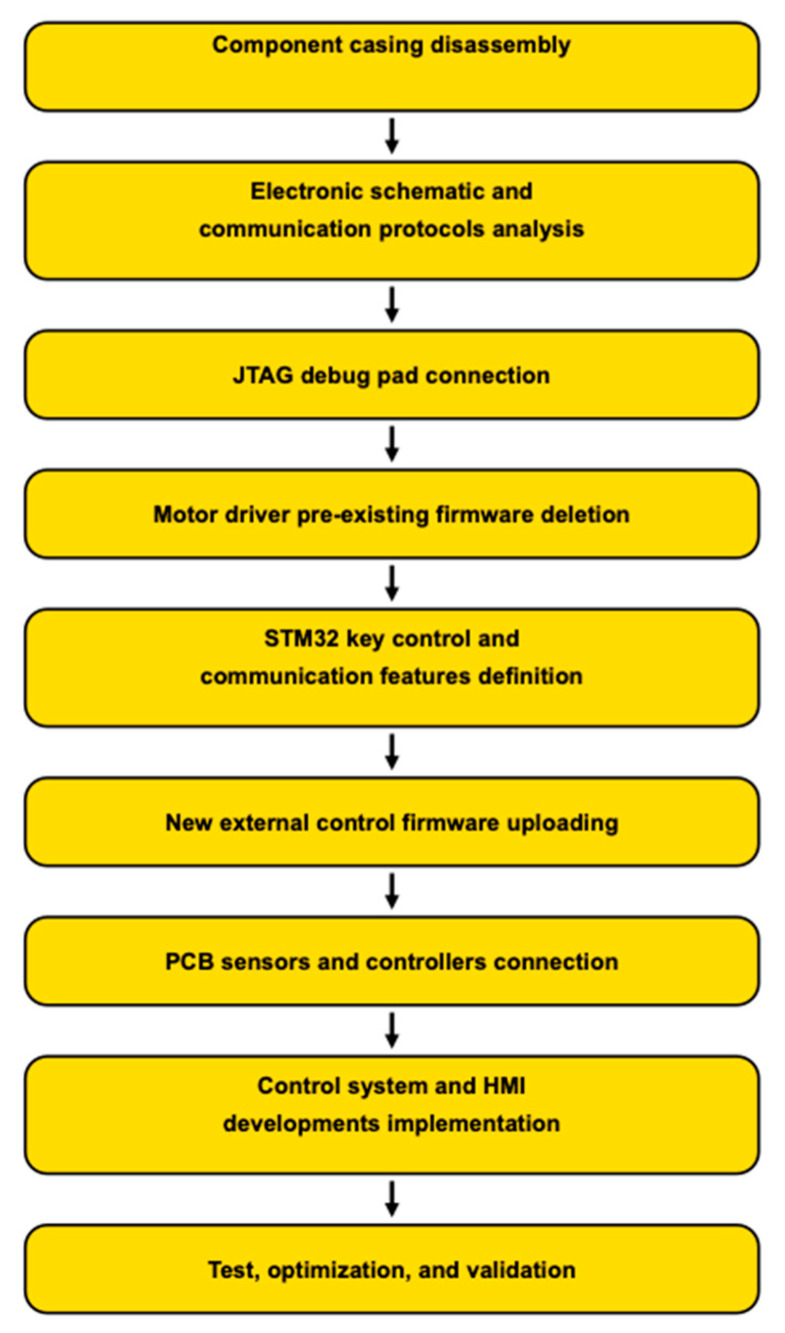
Methodology block diagram.

In order to better understand the methodology used to carry out the reverse engineering approach, we have illustrated it in Figure 2. This shows the main stages, which are detailed in the next sections.

### 2.1. Reprogramming the Motor Controllers

The critical step was modifying the original firmware of the motor driver boards to be able to communicate with the main boards. These boards, equipped with an STM32 type microcontroller (GD32F130C8 from GigaDevice, Beijing, China), are designed to control brushless DC motors based on tilt and acceleration data. Using a commercial debugging tool and firmware flashing software (STM32CubeProgrammer Version 2.19), we were able to access the microcontroller’s memory and analyze its programmed behavior.

By studying the communication protocols in the main control board, the single-wire interface module (SWIM) and JTAG/serial wire debugging (SWD) interfaces were used to communicate with the GD32F130C8 in the TT SD2.2.

This communication allowed the original control program to be bypassed and an external microcontroller, the ESP32 (from Espressif Systems, District of Pudong, Shanghai, China), to be integrated into the system. The new program functions were the measurement of motor information, UART communication, and motor control.

**Figure 3:**
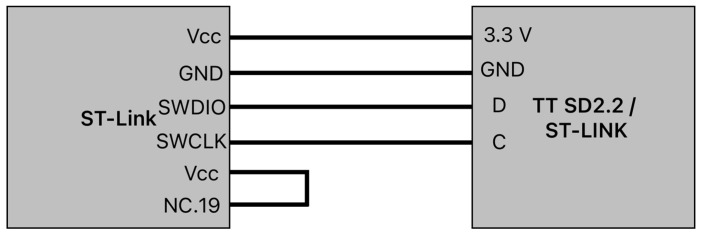
Connection diagram between the programmer and the motor driver board.

The ST-Link debugger (from STMicroelectronics, Geneva, Switzerland) was connected to the motor driver board using an inter-integrated circuit, and the connection diagram is shown in Figure 3. The wiring setup involved the following:

- Power connections: the motor driver board is supplied with 3.3 V thanks to the debugger;
- Signal lines: data ports, such as the clock ports, are connected.

The last connection wired was between the VCC port and the NC.19 port. The reason why this connection is needed is not known, but without it, it will not work. This part is the perfect example of how to test and understand the way flashing the motor driver boards works.

### 2.2. Circuit Redesign

Once the functioning of the hoverboard was understood, it was time to add the software and every other necessary component. To pilot the motor driver boards and allow wireless control, an ESP32 was added to the electronic circuit. This microcontroller was chosen because of its Wi-Fi capacity and its fast work frequency.

The ESP32 was connected to the motor driver boards using UART communication, enabling bidirectional data transfer. A baud rate of 19,200 bps ensured timely transmission for command and feedback from the motor controllers. The wiring setup involved the following:

- Power Connections: The ESP32 was supplied with 5 V from the motor driver board;
- Signal Lines: The transmit (TX) and receive (RX) pins of the ESP32 were connected to the corresponding UART pins on the motor driver board, facilitating the real-time control of the motor parameters.

**Figure 4:**
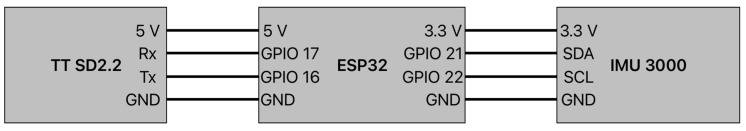
Connection diagram between the ESP32, the IMU 3000, and the motor driver board.

The connections listed above are shown in Figure 4.

Reverse engineering not only allowed the reuse of the hoverboard’s hardware but also provided valuable insights into its operation. These insights informed the design of custom control algorithms and facilitated the seamless integration of new components. By leveraging the existing architecture and adding tailored modifications, we successfully transformed a consumer-grade hoverboard into a functional robotic platform.

**Figure 5:**
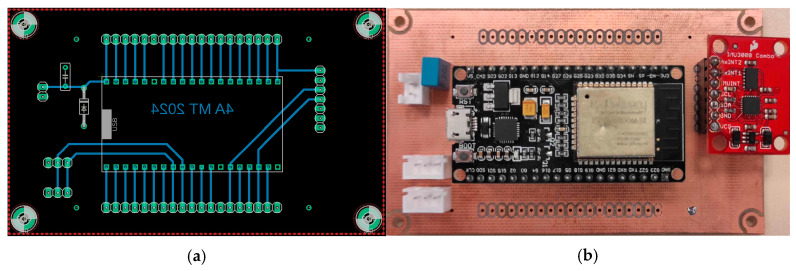
(**a**) PCB schematic, (**b**) PCB photo.

A custom PCB (Printed Circuit Board, see Figure 5) was designed to centralize the connections and reduce the number of wires, ensuring efficient communication and power distribution. This basic board houses the ESP32, the accelerometer, and a small supply circuit to power the sensor and the microcontroller.

### 2.3. Sensor Integration

The IMU (Inertial Measurement Unit), specifically the IMU3000 Combo (from SparkFun Electronics, Boulder, CO, USA), comprises an IMU3000 gyroscope and an ADXL345 accelerometer. It is connected to the ESP32 via the I2C protocol with a 3200 Hz output data rate. The IMU3000 features a three-axis digital gyro that is programmable full-scale up to ±2000 degrees/sec, while the ADXL345 three-axis accelerometer obtains high-resolution measurements up to ±16 g. Similar integrated MEMS-based approaches have previously been demonstrated [10].

The IMU3000 Combo collects raw angular velocity and acceleration data, which are processed in two main steps. First, by calculating the roll and pitch angles from the accelerometer, formulas are derived from the trigonometric relationships between the acceleration components along the axes of a three-axis accelerometer. However, due to rapid direction changes and engine vibrations, this data is very noisy. Second, the angular velocity is integrated to obtain the angular position, but this process drifts over time due to error accumulation in the gyroscope measurements. This is why a Kalman filter is used to combine the gyroscopic measurement with the accelerometer measurement [8,13,14]. This method improves accuracy and reduces noise and drift based on sensor fusion, combining the qualities of gyroscope and accelerometer measurements.

### 2.4. Stabilization and Control System

**Figure 6:**
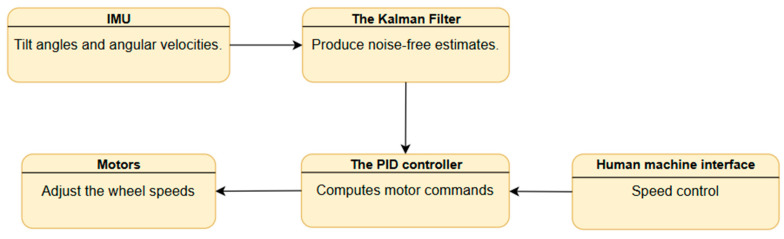
Control loop diagram.

The platform operates on an inverted pendulum, maintaining dynamic stability by aligning wheel motion with the center of gravity. This is why a control loop system was developed, as shown in Figure 6. The system operates by using an IMU (Inertial Measurement Unit) to measure tilt angles and angular velocities. These raw measurements are processed through a Kalman filter, as explained for sensor integration, to reduce noise and provide clean, reliable estimations. The PID controller then takes these estimations and computes commands for the motors, which adjust the wheel speeds to maintain stability and achieve the desired position.

The PID is suitable for controlling the stability of the inverted pendulum by acting through proportional actions, which instantly correct errors to bring the pendulum back to equilibrium, integral actions, which compensate for residual errors by accumulating the error over time, and derivative actions, which anticipate rapid variations to dampen oscillations and avoid instability.

On the other hand, an internet-based application allows for direct position control, ensuring the system responds effectively to user inputs.

### 2.5. Human–Machine Interface

**Figure 7:**
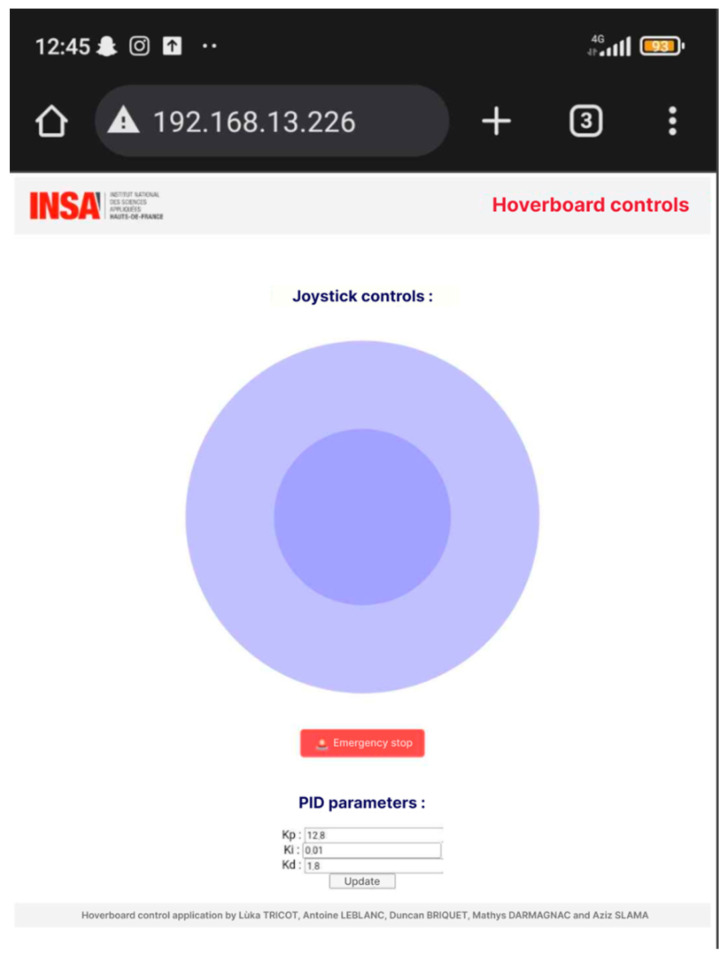
Human–machine interface.

The ESP32 microcontroller serves as a central hub for Wi-Fi communication, acting as a server that hosts a web-based user interface. This interface, accessible via any device with a web browser, features a virtual joystick for controlling the platform’s movement and additional controls for adjusting key parameters such as PID values (illustration in Figure 7).

When the user interacts with the joystick, the interface calculates the directional and speed data, which is normalized and sent to the ESP32 via HTTP requests. The ESP32 processes these requests, translating them into motor commands to achieve precise control. For instance, joystick movements are converted into angle and distance values, which are then used to calculate individual motor speeds based on the desired direction. This approach ensures smooth and responsive navigation.

The interface also includes options for the real-time adjustment of PID parameters. Users can input values for proportional, integral, and derivative gains directly into the interface. These parameters are transmitted to the ESP32, allowing on-the-fly tuning of the stabilization algorithm. This capability is particularly useful for optimizing performance under varying load or terrain conditions.

Emergency stop functionality is another critical feature of the system. The interface includes a dedicated button that sends a command to immediately halt motor activity, ensuring safety in case of unexpected issues.

To enable this functionality, the ESP32 is programmed to handle multiple routes, each corresponding to a specific command, such as updating PID parameters or receiving joystick data. The system leverages asynchronous web server libraries to efficiently manage these requests, minimizing latency and ensuring reliable communication. Additionally, the ESP32 hosts static resources such as HTML, CSS, and JavaScript files using its internal SPIFFS file system, enabling the interface to function without relying on external servers.

This setup provides a robust and flexible communication system, making it possible to control and monitor the platform remotely while maintaining real-time responsiveness.

## 3. Results

The primary objective of this study was to demonstrate two key achievements:

1. Successful reverse engineering of a hoverboard’s original motor driver boards;
2. Effective self-balancing using a PID-based control loop.

This section summarizes the experimental tests conducted to validate both aspects. While the final goal is to enable fully autonomous navigation, such algorithms (e.g., SLAM or path planning) were beyond the scope of the present work. Instead, we concentrated on verifying that the motors could be completely controlled via our custom application and that the platform maintains upright stability under typical small disturbances. The results are organized into the following categories:

### 3.1. Motor Control and Human–Machine Interface (HMI) Testing

To validate the successful reverse engineering of the hoverboard’s motor driver boards, we developed a web-based Human–Machine Interface (HMI) hosted on the ESP32 microcontroller. The user can send target speed and steering commands over Wi-Fi, and the driver boards respond via a UART link at 19,200 bps. We recorded the system’s latency and speed response over a series of repeated commands.

#### 3.1.1. Speed and Power Observations

By reprogramming the TT SD2.2 boards to interface with the ESP32 microcontroller, a reliable communication protocol using UART was established, as described in Section 2.2 Circuit Redesign. This connection enabled precise motor speed and directional control, essential for balancing and motion.

**Figure 8:**
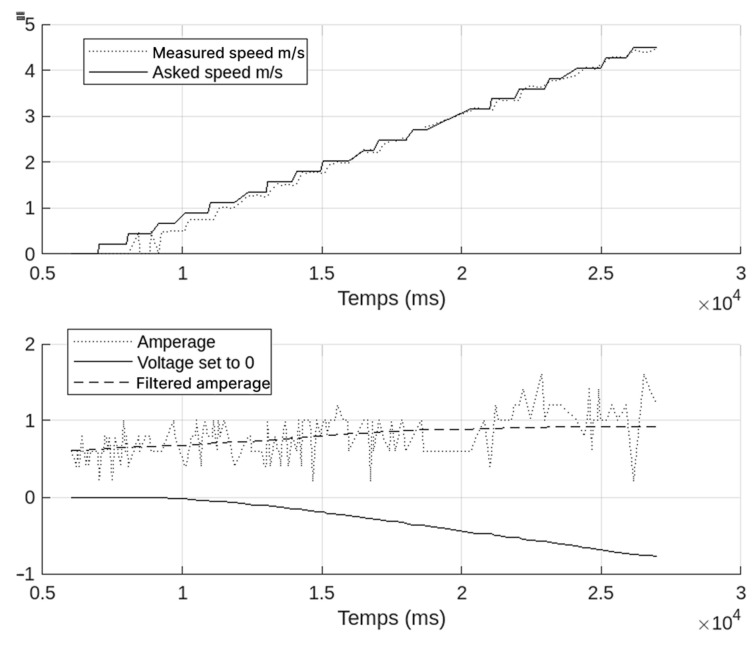
Speed control and power consumption graph.

Figure 8 displays a representative speed control test and the corresponding power consumption over time. We issued step commands from 0% to 50% of the motor’s nominal speed. The voltage and current were monitored through an inline wattmeter.

- Response Time: On average, the motors reached 90% of the target speed in about 0.3 s (±0.05 s).
- Power Consumption: As shown in Figure 8, typical consumption stayed between 20 W and 70 W for moderate speeds, later rising above 100 W under rapid acceleration tests.

These observations confirm that the motor driver boards accurately interpret external commands, a key indicator that the reverse-engineered firmware and communication protocol are functioning correctly. Furthermore, the relatively low power draw suggests that a partially degraded battery can still support teleoperated movement over short sessions.

#### 3.1.2. Reliability of the HMI

We tested command reliability by sending 50 forward/backward instructions in rapid succession. The system logged each command, noting the time to acknowledgment. The average round-trip time was ~40 ms, with zero command losses or misinterpretations.

To ensure user safety, an emergency stop button was included on the HMI interface. It will ensure that the robot is immediately out of service in case of a malfunction or unexpected issue by cutting the power. This button helps prevent potential accidents and ensures a quick response to any situation.

### 3.2. Self-Balancing Under Disturbances

In this section, we quantitatively assess the system’s ability to maintain balance when subjected to common indoor disturbances, such as pushes or uneven flooring. By combining Kalman filter-based sensor fusion and a PID controller, the robot can rapidly detect changes in its tilt angle and apply corrective motor commands to restore equilibrium.

#### 3.2.1. Experimental Setup

To assess the robot’s stabilization performance, we adopted a three-step procedure involving initial conditions, disturbance applications, and sensor fusion with control. First, the hoverboard platform was placed on a flat indoor surface, and the PID controller was initialized at a 0° tilt angle. An additional weight was attached above the original chassis to raise the center of mass, which enhanced the platform’s sensitivity to small pitch changes and thus facilitated more precise PID tuning.

Next, lateral pushes were applied by having an operator briefly exert a moderate force at mid-chassis level. Each push was repeated five times, and the resulting data were recorded at 3200 Hz via the onboard IMU. We defined a “successful recovery” as returning to within ±2° of upright within two seconds of the initial disturbance.

#### 3.2.2. Disturbance Rejection Results

Following the procedure described in Section 3.2.1, we recorded the peak tilt angle and time to recovery for five repeated lateral push tests. Table 1 presents a summary of the experimental data.

Across all trials, the peak tilt ranged between approximately 7° and 8°, with an average time to recovery of ~2.3 s. Only in Trial 5 did a stronger push induce a slightly larger deviation (8.5°), resulting in a delayed response of ~3 s before returning to an upright position. Nevertheless, the system successfully stabilized in all five cases, underscoring the reliability of the Kalman filter-based PID control loop under moderate disturbances.

**Figure 9:**
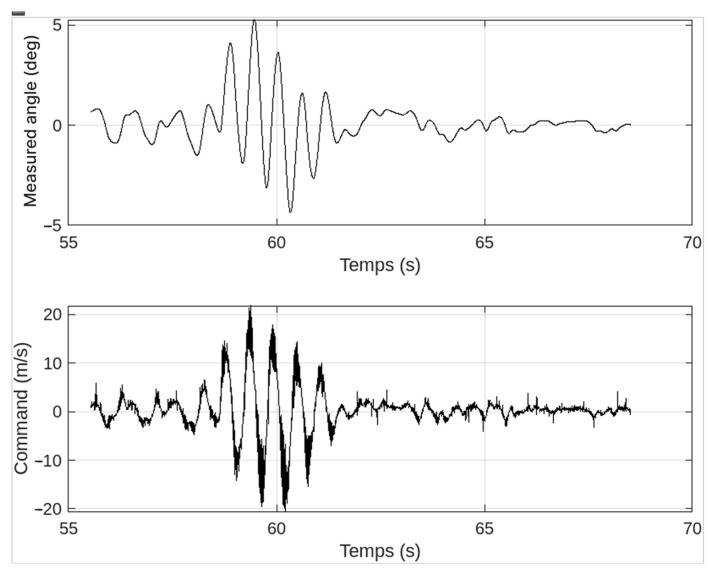
Angle and control in the face of a disturbance.

As illustrated in Figure 9, the angle initially deviates upon disturbance and subsequently oscillates around the equilibrium point. These oscillations gradually diminish, revealing the successful integration of the Kalman filter and the PID controller, which together provide noise reduction and real-time corrective action. By ~2–3 s after the push, the platform consistently returns to within ±2° of vertical, confirming the stability of the control loop even when the battery is partially degraded.

### 3.3. Energy Use and Battery Constraints

Battery aging is a major issue when it comes to recovering second-hand electronics. The initial technical characteristics of the battery proposed by the manufacturer are 4400 mAh 36 V and 2 h 30 min of autonomy, which means an average consumption of 1.76 A.

As shown in Figure 8, the current drawn varies around 1 A, mostly regardless of speed. The lithium-ion battery no longer supports a full human rider; the weight reduction explains the reduction in consumption.

The partial battery degradation is not necessarily prohibitive for short-duration and low-weight robotic tasks [3], and its remaining capacity suffices for lighter loads and intermittent teleoperation.

### 3.4. Discussion

The experimental results demonstrate that reverse-engineered commercial hoverboards can yield a robust two-wheeled platform capable of self-balancing under moderate indoor disturbances. By reprogramming the original motor driver boards, we achieved reliable speed control via Wi-Fi commands and verified that a partially degraded battery remains sufficient for short teleoperated sessions. The disturbance rejection tests, particularly the lateral pushes (Table 1), highlighted the capacity of the Kalman filter-based PID loop to correct tilts of up to ~8.5°, typically restoring the platform to ±2° of upright within 2–3 s.

From an application standpoint, these findings suggest that even worn hoverboard batteries can support small-scale robotic tasks, such as material transport in controlled indoor environments. However, the system showed signs of longer recovery when the disturbance exceeded ~8°, indicating that heavier payloads or larger impacts may require refined PID gains or supplementary mechanical bracing. Additionally, while our power measurements confirm that 30–70 W consumption can be maintained over short intervals, extended operational cycles would likely necessitate a thorough analysis of battery capacity and degradation over time.

Despite these limitations, the project successfully meets its core objectives: (1) proving that hoverboard components can be reverse-engineered and repurposed, and (2) demonstrating stable two-wheeled operation through sensor fusion and PID control. Future efforts can explore alternative filter techniques (e.g., extended or unscented Kalman filters), adaptive control strategies for larger disturbances, and more efficient battery management approaches. Collectively, these upgrades would strengthen the platform’s performance and reliability, paving the way for full autonomy should advanced navigation algorithms be integrated.

## 4. Conclusions

This study highlights the potential of reverse engineering to transform discarded electronic devices, such as commercial hoverboards, into autonomous, cost-effective, modular robotic platforms aimed at sustainability and resource optimization. While the project successfully repurposed hoverboard components to create a functional robotic base, challenges remain in achieving full stability and reliable motion control. To ensure the utility of the mobile base, future iterations could integrate specialized add-on modules, such as motorized arms or shelving units [1,15], to enable advanced material handling tasks in hospital or manufacturing environments. This concept aligns with ongoing research in modular and reconfigurable mobile robots, where a single platform can be quickly adapted to multiple use cases [16].

While the current design relies on a partially worn battery and teleoperated control, our results show that such a configuration is already viable for short-duration tasks where rapid human intervention or remote supervision is acceptable. Looking ahead, integrating autonomous navigation—whether through onboard sensors or external tracking systems—represents a natural extension of this work. Several control strategies have been evaluated in the literature. For instance, Model Predictive Control (MPC) offers an anticipatory control framework that accounts for future system states [17]. Fuzzy logic control, while proficient in handling uncertainties, requires extensive parameter tuning. Moreover, techniques based on Simultaneous Localization and Mapping (SLAM) and reinforcement learning provide promising alternatives for real-time mapping and adaptive decision-making in unstructured environments [18]. The integration of these methodologies is expected to significantly enhance the platform’s performance in complex, dynamic scenarios.

By demonstrating that reverse-engineered hoverboards can serve as low-cost mobile robotic bases, this project highlights a productive route toward reducing e-waste and lowering barriers to entry for advanced robotics. The proposed methodology can be adapted to other personal transport devices, such as electric scooters, broadening its relevance to emerging fields like service robotics, last-mile delivery, and collaborative human–robot interaction. Future research will focus on optimizing control algorithms, integrating advanced navigation sensors, and performing extensive validations under real-world conditions to further improve system performance and reliability.

## Figures and Tables

**Table 1:** Summary of the disturbance rejection results for lateral pushes.

Trial	Peak Tilt (°)	Time to Recovery (s)
Trial 1	7.3	2.5
Trial 2	7.8	2.5
Trial 3	5	2
Trial 4	6.9	2.2
Trial 5	8.5	3

## Data Availability

Data are contained within the article.

## Abbreviations

The following abbreviations are used in this manuscript:

PID	Proportional–Integral–Derivative
HMI	Human–Machine Interface
MPC	Model Predictive Control
BMS	Battery Management System
IMU	Inertial Measurement Unit
SWIM	Single-Wire Interface Module
SWD	Serial Wire Debugging
UART	Universal Asynchronous Receiver–Transmitter
PCB	Printed Circuit Board
I2C	Inter-Integrated Circuit
SLAM	Simultaneous Localization and Mapping
